# A review on human reproductive systems encountering with the severe acute respiratory syndrome coronavirus 2 infection

**DOI:** 10.18502/ijrm.v21i1.12661

**Published:** 2023-02-08

**Authors:** Esmaeil Samadian, Bahman Aghcheli, Roghaye Gharaei, Alijan Tabarraei

**Affiliations:** ^1^Laboratory Sciences Research Center, Golestan University of Medical Sciences, Gorgan, Iran.; ^2^Infection Diseases Research Center, Golestan University of Medical Sciences, Gorgan, Iran.; ^3^Department of Obstetrics and Gynecology, School of Medicine, Golestan University of Medical Sciences, Gorgan, Iran.

**Keywords:** SARS-CoV-2, Human, Reproductive system.

## Abstract

The severe acute respiratory syndrome coronavirus 2 (SARS-CoV2) is the leading cause of the new deadly pneumonia named coronavirus disease 2019 (COVID-19) pandemic. This pathogen has different co-receptors on various tissues, resulting in vast pathophysiological circumstances. Here, we present a comprehensive narrative review focusing on the impact of SARS-CoV2 on human reproduction. Evidence-based literature revealed inconsistent results for this virus in the reproductive organs of patients with COVID-19, even in the critical phase. Conversely, numerous satisfactory data represented those different reproductive activities, from gametogenesis to pregnancy, can be targeted by SARS-CoV2. The severity of COVID-19 depends on the differential expression of the host cellular components required to enter SARS-CoV2. The cytokine storm and oxidative stress coming out during COVID-19 are associated with complications in reproductive endocrinopathies. Men are naturally more susceptible to COVID-19, especially accompanied by orchitis and varicocele. Synergistically the interaction of SARS-CoV2 and female reproductive failures (polycystic ovary syndrome and endometriosis) increases the susceptibility to COVID-19. Thus, pharmaceutical interventions that ameliorate the complications in individuals with reproductive disorders can be helpful to achieve good outcomes in assisted reproductive techniques. Soon, an increase in the infertility rate will likely be an overall impact of SARS-CoV2 in patients who recovered from COVID-19.

## 1. Introduction

The severe acute respiratory syndrome coronavirus 2 (SARS-CoV2) virus, the cause of the coronavirus disease 2019 (COVID-19) pandemic, has been first reported as deadly pneumonia that results in the death of millions of people worldwide (1). The virus's surface protein interacts with angiotensin-converting enzyme (ACE)-2 on the host cell's surface. ACE-2 is the key element of the renin-angiotensin pathway that regulates hemostasis and blood pressure. Although ACE-2 is the main target of this virus, other molecules, such as CD147 (Basigin, BSG), have been suggested as the secondary receptors of severe acute respiratory syndrome 2 (SARS-2) (2). Also, different types of proteases involved in activating S proteins such as furin, transmembrane serine protease 2 (TMPRSS-2) and 4 Cathepsin B and L, and so on are diversely expressed in different tissues of the body (3). This cleavage creates better conformational changes for better fusion and releases the virus genome, leading to higher infectivity (4). However, the ACE-2 receptor is highly expressed in the small intestine, male and female reproductive systems, kidney, heart, and thyroid, moderately expressed in the lung, liver, bladder, colon, and adrenal gland, and slightly in the brain, spleen, bone marrow, and blood cells (5).

In general, COVID-19 has 4 pathological forms: 1) without clinical symptoms with/without detectable virus; 2) with clinical symptoms or with moderate symptoms and the presence of the virus in the body; 3) shaded that the patient has a high titer of the virus; and 4) the recovery state with/without detectable virus (6). The expression of the main target of this virus (ACE-2) varies in different anatomical regions of the male and female reproductive systems (7). Interestingly, numerous studies have suggested that the cytokine storm that emerged in this infectious disease induces undesirable outcomes such as defective spermatogenesis in human reproduction in SARS-2-infected males (8). Age and subsequent changes in sex hormones (estrogen, progesterone, follicle-stimulating hormone (FSH), and luteinizing hormone (LH)), especially in women during their physiological situations (puberty, menstruation, and menopause), and sex (sex chromosome types X or Y, the encoding of immune system-related genes from chromosome X, the high expression of the ACE-2 receptor, the low expression of TMPRSS-2 from chromosome X, the high level of testosterone, the decreased expression of ACE-2, and the elevated level of TMPRSS-2 in men) are the important, impressive factors in COVID-19 (9). The adverse socio-economic conditions due to quarantine, the increased stress and depression, and the importance of population and age distributions lead to huge concerns about the effects of COVID-19 on human reproduction and the infertility rate (10).

In this research, we reviewed the literature on the possible direct and indirect complications induced by SARS-2 in human reproduction. Here, the risk of susceptibility in men and women and patients suffering from common reproductive disorders to the risk of COVID-19 and potential infertility in the presence of SARS-2 are discussed. According to an updated literature review, the putative pathogenesis of SARS-2 infection in human reproductive organs were considered. Also, the reproductive system is not permissive to this viral infection, and the problems caused by COVID-19 can be associated with reproductive failures (10).

## 2. SARS-2 and complications in the male reproductive system

The renin-angiotensin system plays a pivotal role in the regulation of male hormones (testosterone) and the production and concentration of sperms in the testis and epididymis. ACE-2 is differentially expressed in various regions of the male reproductive system. The ACE-2 level is high in Leydig, Sertoli, spermatogonia, prostate epithelial cells, and fibroblast cells. Therefore, these cells are likely to be targeted by SARS-2. In contrast, these targets have low-expression levels in the prostate's hillock area and club cells (11).

Also, ACE-2 is slightly expressed in spermatocytes, spermatids, and other somatic cells. This molecule in Leydig cells modulates the production of testosterone, the production volume of semen, and the health of sperms (11, 12). The highest level of ACE-2 is observed in the age of sex maturation and the high level of males, especially in middle age and high-sexual activity. Low expressions of ACE-2 in sterile men resulted in edema, vacuolization of Sertoli cells, detachment of basal membrane, and destruction of the seminiferous tubule. These events that eventually lead to poor quality in sperms could be due to the inflammation and recruitment of leucocytes and other inflammatory-responding cells in these regions (12).

The high vulnerability of the testes, as well as the elevated inflammatory responses in this organ during orchitis, are important complications in COVID-19 pathogenesis. SARS-2 has deleterious effects on the male reproductive organs by disturbing the autophagy system. The cytokine storm that emerged from this viral infection induces extrinsic and intrinsic apoptotic cascades and DNA damage in the sperm cells (13). SARS-2 affects both the morphology (the number, concentration, and motility) and genetic integrity of the sperm cells. In this regard, the individuals who recovered from COVID-19 with moderate clinical symptoms showed a decrease in the number of sperm cells. Moreover, a more recent systematic review confirmed that the semen samples of patients with moderate SARS-CoV-2 infection had significantly lower sperm concentration, sperm per ejaculation, motile sperm, and progressively motile sperm than normal patients (12).

## 3. SARS-2 in direct or indirect paths?

It is still not clear whether the sex organs such as the testis, epididymis, and sex glands are directly infected by SARS-2 itself or indirectly by the infection of the renal system (14). It is also surprising how this virus reaches the reproductive system. But according to recent reports, these events are more likely to happen through viremia (15). The other possibility is that due to the nature of SARS-2 and the increased temperature of the body during the pathogenesis of COVID-19, the blood/testis barrier can be damaged by these temperature changes, especially in the testis sac (scrotum). Therefore, the direct presence of the virus itself is not needed for the complications in the male reproductive organs. On the other hand, the influence of SARS-2 on different tissues, especially the brain, and uncontrolled inflammation, such as cytokine storm and disturbance in hormone secretion, can contribute to pathological circumstances in human reproductive systems (54-tu). Notably, the LH level in patients with COVID-19 increased statistically in healthy people (16). Meanwhile, the testosterone/LH and FSH/ratios were depleted in these patients. Any imbalance in the sex hormones affects the hypothalamic-pituitary-testis axis and damages the Leydig cells (17). Besides, the decreased level of testosterone with respect to LH, the decreased number or concentration of sperm cells, the decreased volume of semen, the increased fragmented DNA in the sperm cells, and the deregulated sex function in patients with COVID-19 probably represent fundamental damages in the reproductive system resulting from the impacts of SARS-2 on the controlling region of endocrine and sperm production systems in the central nervous system. SARS-2 can pass through the brain/blood barrier and induce lesions in the brain because neural and microglial cells express ACE-2 (18).

This virus induces hormone failures and inflammation in the hypothalamus and gonadotropin systems affecting gonad functions to distress spermatogenesis and the power of male fertility. Although the exact reason for the association between the severe disease and the depleted testosterone level in patients with COVID-19 is unclear, the activation of TMPRSS-2 protease-mediated by this hormone can facilitate the penetrance of SARS-2 (19). More specifically, the presence of proteases such as TMPRSS11B and TMPRSS127 are critical for the infection of this virus in human spermatozoa. Surprisingly, these proteases are present in the proteasomes that can be released from the prostate gland during ejaculation, implying potential pathogenesis of COVID-19 in tissues (20). These events imply possible pathophysiology of COVID-19, which is related to the prostatic tissues. Surprisingly, treatment of prostate cancer with androgen antagonists decreases the risk of COVID-19 due to the immune-modulatory role of androgens, especially in the innate immune system. Also, testosterone can cause differentiation in the T cells, whereas the opposite of this fact has been suggested in COVID-19 patients (21).

However, this sex hormone has anti-inflammatory properties, and the depletion of this hormone in COVID-19 patients caused an increase in the pro-inflammatory cytokines such as gamma-interferon and interleukin-2 (9). Finally, the generation of the immune complexes in the sperm-producing tubules induced inflammation and orchitis in these patients. This inflammatory response and the reactive oxygen species (ROS) generation in these pathological situations damaged the Sertoli cells and disturbed the spermatogenesis process. Additionally, depending on the severity of the disease, the quality of spermatogenesis and the influence of SARS-2 on reproductive functions varied. For example, any change in the temperature of the testis can affect spermatogenesis seriously. The fever generated by the cytokine storms in COVID-19 can cause poor quality in the male gamete (decreasing the motility and the number of sperms in semen) and infertility. Amazingly, individuals with severe symptoms of COVID-19 are more likely to face a secondary cytokine storm named hemophagocytic lymphohistiocytosis. During this deadly reaction with multi-organ failure, these patients showed high-serum ferritins and defective reproductive systems (22).

**Figure 1:**
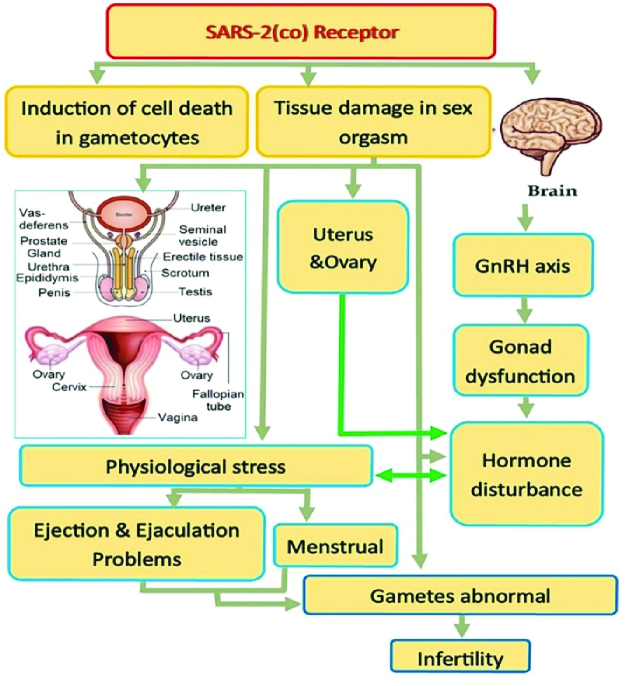
SARS-2 interacts with different organs related to reproductive functions; a pathological mechanism probably proceeded by viremia.

Following the current literature scan, figure 1 summarized the putative interactions of SARS-2 with different organs related to reproductive functions.

## 4. SARS-2 and ROSs generation as a big challenge

The oxygen-free radicals (hydroxyl and superoxide anions, hydrogen peroxide, and so on), generally named ROSs, are physiologically produced by metabolic mediators such as hydrogen peroxide in cellular organelles including mitochondria, endoplasmic reticulum, and peroxisome. ROSs are the necessary mediators in gene expression and regulation, DNA synthesis, cell proliferation, and phosphorylation of cellular proteins. On the other hand, these active molecules deteriorate the cellular components, DNA, lipids, and proteins, and induce cellular death. Therefore, excessive production of these radicals over the power of the antioxidants defensing mechanisms of the body triggers oxidative stress (OS) in different tissues and a serious multi-organ failure. This stress can also induce a necrotic or apoptotic death in the host cells during viral infection. Also, the ROSs can be generated by myeloid-peroxidase, NADPH-oxidase, and inducible nitric oxide (NO) syntheses as immunological activities against pathogens. NO, another type of ROSs, can be produced by macrophages and other leucocytes during viral infections (23).

Interestingly, NO is not very toxic, but when combined with superoxide anions, this combination produces peroxide radicals 1000 times more toxic than free hydroxyl radicals, leading to cellular death (23). In addition, the ROSs promote amplification of the virus depending on the type of the virus and host cells. These molecules also produce pro-inflammatory cytokines, such as tumor necrosis factor alpha (24).

The OS elevates the number of transcription factors such as nuclear factor kappa-light-chain-enhancer of activated B cells and hypoxia-inducible factor-1, increasing the amplification of the virus. SARS-2 induces an uncontrolled OS and inflammatory response. Besides, the interaction between the OS and the immune system has been observed in patients with severe COVID-19 (25). These cross-talks increase the viremia of SARS-2 (26). However, the enzymatic and nonenzymatic anti-oxidative systems, particularly the activity of superoxide dismutase, can improve the quality of semen and fertility. Thus, the ROS generation and OS during the pathophysiology of COVID-19 can probably cause poor quality in sperm cells due to damaging events such as DNA fragmentation and male infertility in these patients (26).

The potential cross-talks between the OS and the cytokine storm in COVID-19 can also be associated with other complications in men. One of the important clinical aspects of COVID-19, especially in patients with severe disease, is lung fibrosis and decreased levels of saturated oxygen. This pathological condition can also lead to depleted production of NO, a mediator necessity for the stiffness and erection of the penis. It is noteworthy that the stiffness of the penis is one of the indicators of the influence of SARS-2 on cardiovascular systems. Surprisingly, individuals with cardiovascular disorders have been considered a high-risk group in the pathogenesis of COVID-19 (27).

**Figure 2:**
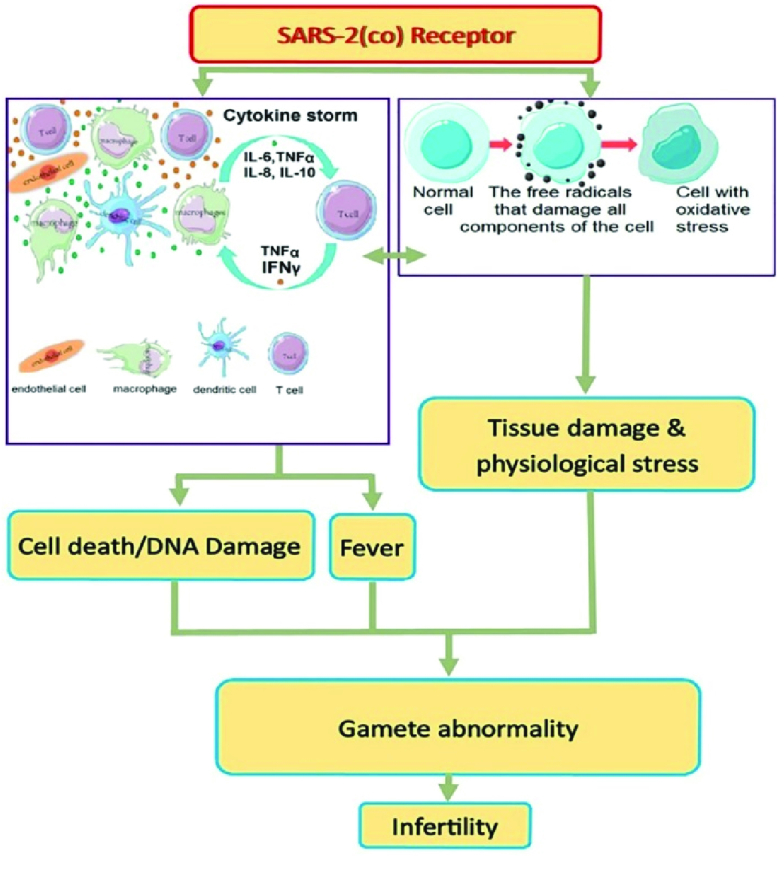
The pathological condition during COVID-19, such as cytokine storm and oxidative stress, affect the reproductive activities as putative pathogeneses of SARS-2.

According to this information, it must be assumed that people with reproductive disorders related to these pathological conditions can be more threatened by SARS-2. For instance, varicocele is the leading cause of the pathological condition of testis and male infertility. The interaction between the OS and the inflammatory cross-talks is important in the pathogenesis of this reproductive failure. Thus, it seems that the risk of COVID-19 and the severity of this infectious disease is higher in men with varicocele. Figure 2 depicted the impacts of cytokine storm and OS produced by SARS-2 on human reproductive systems (27).

## 5. SARS-2 in semen: A conflicting noise

SARS-2 has been detected in many body fluids. This virus is present in the semen in vitro even after 2-3 months of the negativity of the presence of the virus in blood samples. This property of SARS-2 has also been observed in Ebola and Zika viruses. Remarkably, Zika is stable in semen for up to 1 yr. As a result, couples who recovered from COVID-19 are recommended to avoid childbearing for 3 months after negativity of the presence of SARS-2 in their blood samples (28).

On the other hand, the presence of SARS-2 in semen was not confirmed after 1 month of detecting the viral genome in the throat swab specimen (29). Interestingly, the negative presence of SARS-2 was observed in 22-38-yr-old men with severe COVID-19. Similarly, this negative result was also reported in semen even 8 days after the genome of this virus was detected by using the polymerase chain reaction-based method in a case study on a 31-yr-old man. In another report on 20-62-yr-old men, the genome of SARS-2 was not detected in the semen samples after 32 days of the incidence of COVID-19 (30). However, freezing the sex gametes for patients with COVID-19 has been recommended to prevent undesirable problems for the mother and her embryo in the future. Therefore, detecting SARS-2 in semen samples from the patients recovered from COVID-19 has been considered an important challenge for the interest in freezing and storing the semen samples (31).

In this regard, assisted reproductive technologies, such as washing the sperm, can decrease the viral load and the possibility of spreading the virus. Even though there is no established report, detecting SARS-2 in the semen samples of 5% of the patients with severe COVID-19 or the recovering phase of the disease can represent the possibility of infection via sexual means. Therefore, there are conflicting data about the presence of SARS-2 in semen samples of patients with COVID-19 (32). Also, SARS-2 has not been detected in the biopsy samples of testes in men with COVID-1. In contrast, it has been shown that men with COVID-19 have irregular ejaculation. Taken together, although the Center for Disease Control and Prevention has rejected the SARS-2 infection through non-respiratory drops (33), the andrological analysis of the sex glands and quality of the gametocytes in patients with COVID-19 at the age of reproduction must be regarded, especially in the sperm analysis of men recovered from this viral infection. Supporting this issue, we summarized previous investigations on the impact of SARS-Cov2 infection on the male reproductive system in people from different countries. Table I presents evidence showing conflicting data on the presence of SARS-Cov2 in the male reproductive system during the critical phase of COVID-19, particularly in intensive care unit (ICU) patients. As a result, a meta-analysis is needed to further clarify the association between the severity of COVID-19 and complications in the male reproductive system.

**Table 1:** The literature associated the male reproductive system and COVID-19


**Authors, yr (Ref)**	**Country**	**Severity (ICU)**	**Sample size**	**Major findings**
**Rastrelli ** ***et al.*** **, 2021 (34) **	America	RICU	31 COVID-19 cases	Low total testosterone levels were observed in the most severe COVID-19 cases
**Schroeder ** ***et al.*** **, 2021 (35) **	Germany	All patients in ICU	45 cases	Abnormality of sex hormone parameters (dihydrotestosterone and low testosterone levels were shown by most of the men with COVID-19)
**Achua ** ***et al.*** **, 2021 (36) **	USA	ICU	6 COVID-19 cases	The virus was not detected in testicular biopsy
**Hajizadeh Maleki ** ***et al.*** **,** **2021 (37) **	Iran	ICU	Case: 84 Control: 105	SARS-CoV2 can cause male reproductive dysfunction through ROS over-generation pathways activation
**Moghimi ** ***et al.*** **, 2021 (38)**	Iran	ICU	Case: 6 Control: 6	Spermatogenesis was disrupt by COVID-19 via oxidative stress pathway following the induction of apoptosis
**Salciccia ** ***et al.*** **, 2020 (39) **	Italy	20 patients in ICU	29 COVID-19 cases	Abnormality of sex hormone parameters
**Li ** ***et al.*** **, 2020 (8)**	China	6 patient is deceased	Case: 6 Control: 23	The virus was not detected in semen, and orchitis is observed
**Duarte-Neto ** ***et al.*** **, 2020 (40)**	Brazil	7 patients in ICU	10 COVID-19 cases	Orchitis
**Temiz ** ***et al.*** **, 2021 (41)**	Turkey	Unreported	20 COVID-19 cases Control: 10	The virus was not detected in semen and seminal fluid
**Bain ** ***et al.*** **, 2020 (42)**	China	Unreported	37 COVID-19 cases	The virus was detected in testicular tissues
**Yang ** ***et al.*** **, 2020 (43)**	China	Unreported	12 COVID-19 Post-mortem cases	The virus was detected in testicular tissues
**Li ** ***et al.*** **, 2020 (44)**	China	Unreported	38 cases	The virus was detected in semen
**Pan ** ***et al.*** **, 2020 (45)**	China	Unreported	34 COVID-19 cases	The virus was not detected in semen
**Ma ** ***et al.*** **, 2020 (46)**	China	Unreported	12 COVID-19 cases	The virus was not detected in semen, but semen and sex-hormone parameters are abnormal
**Holtmann ** ***et al.*** **, 2020 (47)**	Germany	Unreported	18COVID-19 cases Control: 14	The virus was not detected in semen, but the semen parameter is abnormal
**Guo ** ***et al.*** **, 2021 (30) **	China	Unreported	23 COVID-19 cases	The virus was not detected in semen
**Ning ** ***et al.*** **, 2020 (48)**	China	Unreported	17 COVID-19 cases and one post-mortem	The virus was not detected in semen and seminal fluid
**Pavone ** ***et al.*** **, 2020 (49)**	Italy	Unreported	9 COVID-19 cases	Despite of prolonged nasopharyngeal swab positivity, no evidence of SARS-CoV2 was found in semen
**Ma ** ***et al.*** **, 2021 (46)**	China	Unreported	81 COVID-19 cases	SARS-CoV2 may influence the production of LH, FSH, and testosterone as well as the testes
ICU: Intensive care unit, RICU: Respiratory intensive care unit, COVID-19: Coronavirus disease 2019, SARS-CoV2: Severe acute respiratory syndrome coronavirus 2, LH: Luteinizing hormone, FSH: Follicle-stimulating hormone				

## 6. Putative mechanisms of men's high susceptibility to COVID-19

There is convincing evidence that the risk of COVID-19 in men is higher than in women, without considering their age. In this respect, many men with COVID-19, particularly with a severe form of the disease, showed a pattern of alopecia related to the high level of androgens. Far from the fact that men's exposure to SARS-2 is higher than women's, it has also been suggested that the high level of androgens and consequently the elevated expression of ACE-2 in men increase the risk of COVID-19. From a mechanistic point of view, the intricate balance between ACE-2 and androgen levels plays a critical role in the risk of this infection. The ACE-2 and androgen receptors are located on the short arm of chromosome X that can escape the inactivation mechanisms in the barr body (50). Also, the endocrinal changes can cause hypo-methylation and increased expression of ACE-2. Alternatively, the androgen produced from the testis binds its receptor (androgen response element) in the vicinity of the TMPRSS-2 promoter region and increases the expression of TMPRSS-2. These events can increase the death rate and severity of COVID-19 in men (51).

## 7. Sensitivity of female reproductive system to SARS-CoV2 infection

Because of endocrine changes in the female reproductive system, the expression of ACE-2 and its proteases varies during the different menstrual cycles (puberty, pregnancy, and menopause). Therefore, the possible risks for SARS-2 infection are different during these periods, as shown in figure 1 (52). The ovary and sex hormone-producing cells, such as theca cells, display a high level of ACE-2 (53). In the uterus, this receptor is highly expressed in the epithelial and stromal cells of the endometrium and myometrium tissues. Interestingly, the SARS-2 genome was not detected in oocytes (54). It seems that the oocytes show no distinct level of TMPRSS-2. These protease-negative cells can be infected by SARS-2 through the infected sperm cells. SARS-2 also employs other proteases, such as Cathepsin B and CO-2, with moderate to high expression levels in the endometrium. Myxovirus resistance protein 1 (MX-1) recruits neutrophils in the infectious tissues and helps with the release of elastase from these neutrophils and the easier and better cleavage of ACE-W by TMPRSS-2. Surprisingly, the simultaneous contribution of MX-1 and TMPRSS-4 can amplify the severity of COVID-19 during pregnancy. In addition, the expressions of furin, CTSV, TMPRSS-4, and MX-1 during the primary and secondary phases of the menstrual cycle are impressive potential factors in the risk of SARS-2 infection in females (52). Furin is another key protease required for the infection of this virus.

This protease is significantly associated with basigin, the secondary receptor of SARS-2 (55). Furin, without the involvement of other proteases, can cut the surface protein of SARS-2 attached to BSG. Alternatively, women older than 50 are shown to be more sensitive to COVID-19 during the primary phase of their menstrual cycle. In contrast, as women's age increases, the expression of TMPRSS-2 in them can decrease during the secondary and late phases of menstrual cycles. Also, the level of ACE-2 and the production of estrogen and androgen can be depleted as age increases, without considering gender. This can indicate a decrease in the penetrance of SARS-2 as age increases. However, SARS-2 can affect the balance of effective hormones in the endometrium through the expression of ACE-2. The increased level of ACE-2 in patients with endometrial cancer was highly associated with the risk of COVID-19. The estrogen produced by the ovary leads to an increase in ACE-2 and depletion of TMPRSS-2.

Conversely, androgens can activate this protease. Since the levels of androgens are normally low in women, androgens play a protective role in this group of people (56). Supportedly, we listed the literature related to the impact of SARS-Cov2 on different functions of the female reproductive system. Table II provides evidence for the complications in women with COVID-19, particularly in ICU.

**Table 2:** The literature associated with the female reproductive system and COVID-19


**Authors, yr (Ref)**	**Country**	**Severity (ICU)**	**Sample size**	**Major findings**
**Qiu ** ***et al.*** **, 2020 (57)**	China	All patients in ICU	10 COVID-19 cases	Absence of SARS-CoV2 in the vaginal fluid, cervical smear, and oocytes
**Liao ** ***et al.*** **, 2020 (58)**	China	ICU	Case: 10 pregnant women confirmed covid19 Control: 53 pregnant women	No difference was reported about maternal (postpartum hemorrhage, gestational age and perinatal resection rates) and neonatal results (neonatal asphyxia rates and birth weight) in COVID-19 patients
**Futterman ** ***et al.*** **, 2020 (59)**	New York	ICU	2 cases gestation with suspected COVID-19	1 patient (22 wk of gestation) experienced intrauterine fetal death. 1 patient (29 wk of gestation) for no fetal reasoning status experienced emergency cesarean delivery
**Cooke ** ***et al.*** **, 2020 (60)**	UK	ICU	2 cases of SARS-CoV2 infection in very preterm pregnancy	COVID-19 infection was not reported in newborn infants
**González Romero ** ***et al.*** **,** **2020 (61)**	Spain	ICU	One woman case	COVID-19 infection was not reported in newborn infants
**Sentilhes ** ***et al.*** **, 2020 (62)**	France	ICU (N;13)	54 cases of pregnant women; confirmed (n = 38) and suspected (n = 16) coronavirus disease	3 out of 21 pregnant women experienced preterm deliveries as the maternal respiratory complications
**Knight ** ***et al.*** **, 2020 (63)**	UK	ICU (N;41)	427 cases of pregnant women with confirmed SARS-CoV-2 infection	5% of the infants had COVID-19 infection
**Li ** ***et al.*** **, 2020 (64)**	China	NICU	16 cases of pregnant women confirmed COVID-19	Meaningfully higher preterm delivery was reported as result of maternal complications in patients with COVID-19. No detection of COVID-19 infection in newborn infants and none developed neonatal complications
**Govind ** ***et al.*** **, 2020 (65)**	UK	Emergency CS	9 women cases	SARS-CoV-2 RNA was reported in1out of 9 newborns
**Khan ** ***et al.*** **, 2020 (66)**	China	CS	17 cases of pregnant women infected with COVID-19	Complications were shown by 5 out of 17 pregnant women. All of these cases required cesarean section. 2 out of 17 newborns were positive for the SARS-CoV-2 RNA, and 5 out of 17 revealed neonatal pneumonia
ICU: Intensive care unit, COVID-19: Coronavirus disease 2019, SARS-CoV2: Severe acute respiratory syndrome coronavirus 2, NICU: Newborn intensive care unit, CS: Cesarean section				

## 8. Women with reproductive failures: The high-risk groups

The increased stress and disturbance of the intricate balance in sex hormones can cause OS, and in the female reproductive system, they can be associated with infertility. These pathological circumstances (Figures 1, and 2) can be typically observed in gynecological disorders such as polycystic ovary syndrome (PCOS) and endometriosis. PCOS is the most common cause of infertility in women at the age of reproduction (67).

Different endocrinal-inflammatory cross-talks are involved in forming a polycystic ovary. The excessive production of ROS and pro-inflammatory cytokines, including tumor necrosis factor - α , IL-1b, IL-2, 6, 7, and 8, has been found in women with PCOS. Accordingly, women with PCOS are likely to show a more severe form of COVID-19 due to their similar pathology. Of course, other factors, such as an increase in serum prolactin, depletion of thyroid hormones, hereditary nonclassical adrenal hyperplasia, hyperandrogenism, and hirsutism, increase the risk of COVID-19 in women with PCOS (68).

Importantly, an increase in androgens can contribute to the risks of PCOS and COVID-19 in these women by activating macrophages and neutrophils and producing inflammatory cytokines (69). However, the anti-inflammatory role of the female sex hormones (estrogen and progesterone) results in the women's resistance to COVID-19. In contrary to androgens, estrogen can prevent the migration of inflammatory cells to the infectious tissue, induce anti-inflammatory cytokines such as IL-4 and IL-10, and activates T helper-2 and plasma cells to produce antibodies (70). In other words, a high estrogen level decreases the expression of ACE-2 in women and, consequently, the risk of SARS-2 infection. Hence, the application of estrogen and progesterone can decrease the complications of COVID-19 in women. However, the complications in women with PCOS, including obesity, hypertension, diabetes type 2, metabolic syndrome, non-alcoholic fatty liver, cardiovascular diseases, hyperactivity of the immune system, hyperandrogenism, and insufficient vitamin D, increases their susceptibility to COVID-19 (69).

**Figure 3:**
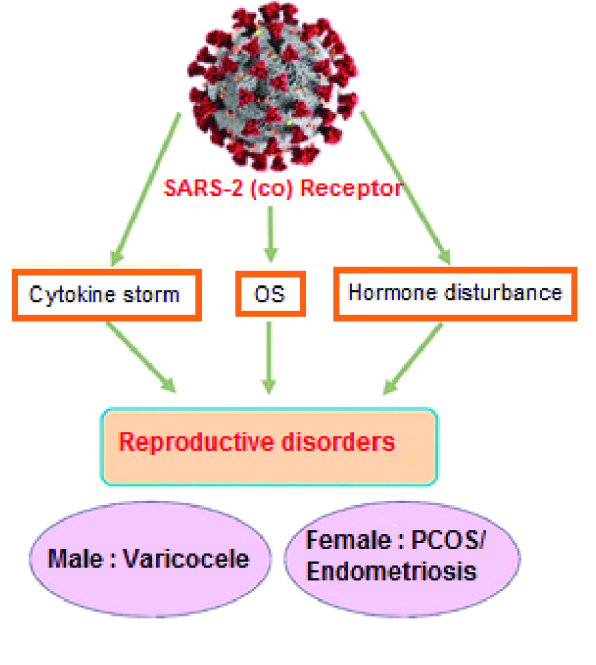
The interrelations between COVID-19 and human reproductive disorders. The involvement of inflammatory responses, OS, and deregulated endocrine systems in both COVID-19 and reproductive failures can increase the risk of SARS-2 infection and complications in individuals with reproductive abnormalities.

As promising therapeutic interventions, the administration of vitamin D and androgen antagonists can ameliorate testosterone and C-reactive protein levels and increase antioxidants, and remove the complexities observed in both PCOS and COVID-19. On the other hand, racial and ethnic differences are big challenges for a good conclusion (71). Unlike PCOS patients, women with endometriosis have not been considered high-risk people in SARS-2 infection groups. Endometriosis is benign pathophysiological damage with an unknown etiology in women at the age of reproduction. The complexity and asymptomatic features of this chronic disease can delay its diagnosis, and the patient is most likely to have this disease for 10 yr, even before diagnosis (72). Endometriosis induces the recruitment of leucocytes, especially the cell communities involved in the innate immune response and cellular swelling. Although the type and position of pathological lesions in endometriosis differ from COVID-19, and this reproductive disease cannot increase these women's susceptibility to COVID-19, the involvement of inflammatory cross-talks in endometriosis can amplify the complications of SARS-2 infection in these women. Therefore, these interactions must not be neglected, and new pharmaceutical guidelines are essential to be considered for patients with reproductive disorders throughout the pathological conditions in SARS-2 infection (73). As depicted in figure 3, the common pathological interrelations between COVID-19 and human reproductive disorders are raising alarms about the risk and severity of this viral infection in men and women with reproductive disorders.

## 9. Conclusion

Different aspects of COVID-19 are still unclear in human reproductive biology. The reproductive system is sensitive to SARS-2, and this virus can directly affect its physiological activities. Even if this system is not permissive for this viral infection, the problems caused by COVID-19 are associated with reproductive failures, implying indirect interactions between SARS-2 and the reproductive system. Numerous distinctions affect the expressions of the receptors and proteases required for SARS-2 infection and consequently, the pathological results. High levels of androgens and ACE-2, and activation of TMPRSS-2 in the normal physiology of the male reproductive system naturally makes men a high-risk group in terms of the SARS-2 infection. The interaction between the OS and the inflammatory cross-talks in reproductive disorders (varicocele in men and PCOS and endometriosis in women) can cause these patients to become more risky groups in terms of being infected by COVID-19. These complications can also increase the severity of this infectious disease. Nevertheless, the excessive production of ROS and inflammatory cytokines during the pathogenesis of COVID-19 can multiply the complications in individuals with these reproductive failures and increase the infertility rate in patients recovering from COVID-19. Therefore, administering anti-androgens, estrogen agonists, and vitamin D and enrichment of oxidative scavengers in these patients can be an effective therapeutic strategy for antagonizing the complications in both SARS-2 infection and reproductive disorders.

##  Conflict of Interest

The authors do not have any affiliation beside any organization as an indirect or direct financial contribution the subject matter mentioned in the manuscript.

All authors have taken part in (1) conception design and conception, or analysis and elucidation of the data; (2) article drafting or revising it in critical manner for significant intellectual content; also (3) approval of the last version.

We have not submitted this manuscript yet, furthermore it is not under review with another journal or publishing venue.
